# Maternal and fetal outcomes in 151 cases of thrombocytopenia in pregnancy

**DOI:** 10.3389/fcell.2025.1608647

**Published:** 2025-08-29

**Authors:** Chinwe Oluchi-Amaka Ibeh, Feng Guo, Xiuhua Yang

**Affiliations:** ^1^ Department of Obstetrics, The First Hospital of China Medical University, Shenyang, China; ^2^ Department of Emergency Medicine, Shengjing Hospital of China Medical University, Shenyang, China

**Keywords:** pregnancy, thrombocytopenia, hypertensive disorders in pregnancy, immune thrombocytopenia, postpartum hemorrhage

## Abstract

**Introduction:**

Thrombocytopenia during pregnancy is one of the important causes of maternal and perinatal mortality. This study aims to retrospectively analyze the clinical data of 151 pregnant patients with thrombocytopenia, in order to help obstetricians better understand the etiology, related risk factors and maternal and fetal outcomes of this disease.

**Methods:**

A total of 151 cases of pregnant women with thrombocytopenia were collected. According to the cause of thrombocytopenia, patients were divided into gestational thrombocytopenia (GT) group, hypertensive disorders in pregnancy (HDP) group, immune thrombocytopenia (ITP) group and the other group. According to the degree of thrombocytopenia, patients were divided into mild group, moderate group and severe group. According to different grouping criteria, the clinical characteristics, delivery outcomes and delivery modes, maternal treatments during pregnancy, maternal laboratory indexes, and neonatal birth conditions were compared.

**Results:**

Among the 151 patients, the GT group had the largest proportion. Moreover, the ITP group had a higher proportion of skin and mucous membrane bleeding during pregnancy, the smallest gestational age at first diagnosis and the lowest platelet count at first diagnosis. The treatment effect of glucocorticoids alone in the ITP group was not good. The HDP group had a higher neonatal intensive care unit (NICU) transfer rate and the lowest birth weight in newborns. In terms of severity, majority of the patients were in the mild group. The parameters of thromboelastography (TEG) were related to the pre-delivery platelet count of patients in the moderate and severe groups, but not in the mild group.

**Conclusion:**

In conclusion, ITP is associated with more severe thrombocytopenia and bleeding, often presenting in the early stage of pregnancy. In the treatment of ITP, the combined use of glucocorticoids and platelet transfusion is recommended. TEG parameter analysis suggests that patients in the moderate and severe groups may have changes in the blood coagulation and fibrinolysis systems. More attention should be paid to the monitoring of the newborns delivered by HDP patients.

## 1 Introduction

Thrombocytopenia during pregnancy is a relatively common disease in the gestational period, involving about 8%–10% of low-risk pregnant women (Townsley, 2013). Generally, thrombocytopenia is considered to be the case where the platelet count is less than 150 × 10^9^/L (Kam et al., 2004; Smock and Perkins, 2014; Veneri et al., 2009). However, only platelet counts below 100 × 10^9^/L are considered clinical significant (Smock and Perkins, 2014; Veneri et al., 2009). Thrombocytopenia is caused by increased destruction or reduced production of platelets (Kam et al., 2004). During pregnancy, the physiological system of pregnant women undergoes changes that alters the concentration of plasma coagulation factors and blood system components (Bar et al., 2025; Wang et al., 2021). Some secondary physiological changes specific to pregnancy, such as increased blood volume, abnormal platelet activation and increased platelet clearance rate, may eventually cause thrombocytopenia (Townsley, 2013). Patients with thrombocytopenia during pregnancy may show bleeding symptoms during physical examination such as bruising, petechiae, purpura, oral mucosal blood blisters and conjunctival hemorrhages (Fogerty and Kuter, 2024).

According to the severity of the disease, some scholars believe that platelet count of 100–150 × 10^9^/L is mild thrombocytopenia, 50–100 × 10^9^/L is moderate thrombocytopenia, and less than 50 × 10^9^/L is severe thrombocytopenia (Kam et al., 2004). In severe thrombocytopenia, life-threatening bleeding may occur, which is manifested by pulmonary bleeding, gastrointestinal bleeding and rare intracranial hemorrhage (Connors and Fein, 2023; Mithoowani et al., 2020). Thrombocytopenia in pregnancy is also related to the occurrence of premature birth (Parnas et al., 2006). Patients with platelet count <20 × 10^9^/L have the risk of spontaneous intracranial hemorrhage, postpartum hemorrhage and placental abruption. In severe cases, disseminated intravascular coagulation (DIC) may occur and cause serious impact on the health of mothers and fetuses (Kelton, 2002).

The etiology of thrombocytopenia in pregnancy can be classified according to “pregnancy specific” etiology and “general etiology.” The etiology of thrombocytopenia in pregnancy may include gestational thrombocytopenia (GT), pre-eclampsia (PE) and hypertensive disorders in pregnancy (HDP) associated thrombocytopenia caused by HELLP syndrome (hemolysis, elevated liver enzymes and thrombocytopenia), immune thrombocytopenia (ITP), hereditary thrombocytopenia Type 2B von Willebrand disease, drug-induced thrombocytopenia, infection, liver cirrhosis, splenomegaly, bone marrow diseases (such as aplastic anemia, myelodysplastic syndrome, leukemia, and lymphoma), paroxysmal nocturnal hemoglobinuria, complement mediated thrombotic microangiopathy, thrombotic thrombocytopenic purpura (TTP), and autoimmune diseases (such as lupus erythematosus, antiphospholipid syndrome (APS)) (Pishko and Marshall, 2022).

The most common cause of thrombocytopenia in pregnancy is GT (Yan et al., 2016; Park, 2022; Fogerty, 2018), accounting for about 75% of all thrombocytopenia in pregnancies (Parnas et al., 2006; Fogerty, 2018; Yuce et al., 2014) and 5%–11% of all pregnancies (Cines and Levine, 2017a). Its symptoms are usually relatively mild, rarely posing a serious threat to the safety of the mother and fetus (Townsley, 2013; Fogerty and Kuter, 2024; Rottenstreich et al., 2018), and often occurring in the third trimester of pregnancy (Fogerty and Kuter, 2024; Cines and Levine, 2017b; Reese et al., 2018). It is worth noting that few cases of moderate to severe thrombocytopenia could be caused by GT, therefore, before making a diagnosis of GT in these cases, a comprehensive clinical evaluation should be conducted, and other potential causes should be examined (Cines and Levine, 2017a). Mild thrombocytopenia, especially when the platelet count ≥70 × 10^9^/L, strongly suggests the diagnosis of GT. For GT patients with platelet count <80 × 10^9^/L, platelet count examination should be performed on the first and fourth day after birth (Gernsheimer et al., 2013).

HDP caused by PE and HELLP syndrome is also a common cause of thrombocytopenia in the second and third trimester of pregnancy, accounting for about 21% (Kam et al., 2004; Parnas et al., 2006; Yuce et al., 2014). Platelet <100 × 10^9^/L is one of the diagnostic criteria for severe PE (Mol et al., 2016). HELLP syndrome is a slightly different PE, characterized by more severe thrombocytopenia (Cines and Levine, 2017b) and higher maternal and neonatal mortality (Young et al., 2010). Patients with HELLP syndrome have a higher rate of cesarean section, and may also have placental abruption and DIC, which may require blood transfusion, and prolong the length of hospital stay (Young et al., 2010). When patients with hypertensive disorders in pregnancy have progressive thrombocytopenia, the diagnosis of HELLP syndrome should be considered. DIC occurs in 20% of HELLP syndrome, which can lead to uncontrollable massive bleeding (Cines and Levine, 2017b; Brown et al., 2018; Fitzpatrick et al., 2014; Thomas et al., 2016).

ITP is another cause of thrombocytopenia in pregnancy, which can occur in any of the different trimesters of pregnancy (Fogerty and Kuter, 2024; Pishko and Marshall, 2022; Park, 2022) and even postpartum (Fogerty and Kuter, 2024), accounting for 3%–10% of thrombocytopenia during pregnancy (Fogerty and Kuter, 2024; Parnas et al., 2006; Yuce et al., 2014). The platelet count of ITP is significantly lower than that of GT. However, there is still no gold standard method to distinguish GT and ITP (Fogerty and Kuter, 2024; Cines and Levine, 2017a). If the platelet count drops below 80 × 10^9^/L during pregnancy, the possibility of ITP should be considered (Townsley, 2013; Cines and Levine, 2017b). A guideline on ITP points out that if the platelet count is 20–30 × 10^9^/L and there is no active bleeding, most pregnancies are safe, and it is safer to have the platelet count ≥50 × 10^9^/L during delivery (Provan et al., 2019).

TTP is a rare life-threatening hematological disease (Thomas et al., 2016; Joly et al., 2017), characterized by widespread blood vessel clotting and bleeding (Xu et al., 2024), microangiopathic hemolytic anemia, severe thrombocytopenia, and organ ischemia linked to disseminated microvascular platelet rich-thrombi (Joly et al., 2017). It presents in any trimester or postpartum (Fogerty and Kuter, 2024; Martin et al., 2008) and in about 5%–25% of TTP cases, pregnancy may be a pathogenic factor (Gerth et al., 2009). The symptoms of TTP are like those of thrombocytopenia associated with severe PE and HELLP syndrome and hemolytic uremic syndrome, so it needs to be differentiated (Xu et al., 2024). When pregnant women with thrombotic micro angiopathies do not meet the diagnostic criteria for severe PE or HELLP syndrome, if the platelet count drops below 20 × 10^9^/L, or if neurological symptoms occur, such as numbness, aphasia, etc., the possibility of TTP should be considered (Martin et al., 2008; George et al., 2015). In late diagnosis and untreated TTP, the mortality rate can go as high as 90% (Xu et al., 2024; Zhou et al., 2017) and microvascular thrombosis leading to fetal growth restriction and/or fetal death, may develop due to impaired placental circulation (Ferrari and Peyvandi, 2020). If the maternal platelet count does not recover to more than 100 × 10^9^/L within 48–72 h after delivery, and the clinical signs and symptoms are not relieved, then the diagnosis of TTP should be considered (George et al., 2015).

APS is an autoimmune disease characterized by arterial or venous thrombosis and/or pregnancy complications (Jin et al., 2022). Thrombocytopenia is a common blood system manifestation in patients with APS, with an incidence of 16%–53% (Cervera et al., 2011), and its mechanism may be due to platelet consumption and/or destruction mediated by antiphospholipid antibodies (Vreede et al., 2019). APS is associated with the increased incidence of unexplained recurrent abortion, fetal growth restriction, premature birth, stillbirth, neonatal death, early-onset PE and severe PE (Park, 2022; De Carolis et al., 2018; Liu and Sun, 2019).

Therefore, thrombocytopenia in pregnancy is an important reason for the increase of maternal and perinatal mortality (Huang et al., 2020). Maternal thrombocytopenia may cause severe postpartum hemorrhage and even require hysterectomy. For pregnant women whose platelet count is lower than 50 × 10^9^/L, these patients may occasionally require intravenous immunoglobulin (IVIg) to maintain safe platelet counts throughout pregnancy or especially in preparation for delivery when a rapid platelet increase is required, as platelet count greater than 50 × 10^9^/L is preferred for delivery (Provan et al., 2019). The newborns of pregnant women with thrombocytopenia may have adverse consequences such as premature delivery, neonatal thrombocytopenia (Guillet et al., 2023; van der Lugt et al., 2013), neonatal asphyxia (McCrae, 2010; Wang et al., 2017) and intracranial hemorrhage (Cines and Levine, 2017b; Li et al., 2022). Some independent predictors of thrombocytopenia include poor economic conditions, elderly mothers, alcohol consumption and human immunodeficiency virus (HIV) infection (Haile et al., 2022).

In this study, we retrospectively analyzed the clinical data of 151 cases of pregnancy with thrombocytopenia. According to the etiological classification and disease severity, patients were divided into different groups, and the clinical characteristics, delivery outcomes and delivery modes, maternal treatments during pregnancy, maternal laboratory indexes, and neonatal birth conditions of each group were compared. The objective of this manuscript is to help obstetricians better understand the etiology, related risk factors and maternal and fetal outcomes of this disease and improve the prognosis by increasing related monitoring.

## 2 Materials and methods

### 2.1 Study population

A total of 151 pregnant women diagnosed with thrombocytopenia aged 18–40 years old, who gave birth in our obstetric department from December 2010 to July 2024 were collected. Two or more occurrences of platelet count less than 100 × 10^9^/L during pregnancy are considered thrombocytopenia. Patients with twin or triple pregnancies, thrombocytopenia caused by medication or viral infections, and congenital coagulation disorders were excluded. Our study was approved by the Medical Science Research Ethics Committee of the First Hospital of China Medical University (Approval No. 2021-108).

According to the cause of thrombocytopenia, patients were divided into gestational thrombocytopenia (GT) group, hypertensive disorders in pregnancy (HDP) group, immune thrombocytopenia (ITP) group and the other group. The GT group includes cases where thrombocytopenia first occurs during pregnancy without history of thrombocytopenia before pregnancy, and platelet counts typically recover spontaneously after delivery, usually without causing significant maternal or fetal complications. When diagnosing GT, it is necessary to exclude other diseases that cause thrombocytopenia. The HDP group comprises pregnant women with thrombocytopenia associated with gestational hypertension, particularly those with PE or HELLP syndrome. The ITP group involves pregnant women with evident bleeding symptoms, such as bleeding points and bruising on the skin and mucous membranes, or symptoms of visceral bleeding, with a significant decrease in platelet counts. Bone marrow examination in ITP cases shows normal or increased megakaryocytes with maturation disorders. The other group includes thrombocytopenia during pregnancy caused by other conditions, such as TTP, aplastic anemia, systemic lupus erythematosus, APS, Sjögren’s syndrome, undifferentiated connective tissue disease, myelodysplastic syndrome, or hereditary thrombocytopenia.

According to the degree of thrombocytopenia (Veneri et al., 2009), patients were divided into mild group (platelet: 50–100 × 10^9^/L), moderate group (platelet: 30–50 × 10^9^/L) and severe group (platelet: <30 × 10^9^/L).

### 2.2 Observed indicators

Clinical records were reviewed to collect the following information: the clinical characteristics of each group of patients (age, skin and mucosa bleeding during pregnancy, gestational age at first diagnosis, platelet count at first diagnosis, lowest platelet count during pregnancy, and platelet count on the third day after delivery), whether the patient was transferred to intensive care unit (ICU), length of hospital stay, gestational age at delivery, and the occurrence of postpartum hemorrhage), and delivery mode. Postpartum hemorrhage was defined as the loss of 500 mL or more of blood with a vaginal delivery or 1,000 mL or more with a caesarean section. Maternal treatments for thrombocytopenia during pregnancy included the use of glucocorticoids, platelet transfusions, or IVIg. The criteria for determining the effectiveness of treatment are platelet count ≥5 × 10^9^/L within 24–96 h after treatment, otherwise it is considered ineffective (Provan et al., 2019; Bauer et al., 2021; Estcourt et al., 2017).

We collected the results of blood routine test within 1 week before delivery and on the third day after delivery at our hospital. Blood routine indicators included platelets, hemoglobin (Hb), platelet distribution width (PDW), mean platelet volume (MPV), mean platelet count (PCT), and platelet large cell ratio (P-LCR). Coagulation function indicators included prothrombin time (PT), activated partial thromboplastin time (APTT), D-dimer, international normalized ratio (INR), coagulation time (TT), and plasma fibrinogen (Fg). Thromboelastography (TEG) parameters included reaction time (R), kinetics (K), rate of blood clot formation (Angle), maximum amplitude (MA), clot lysis at 30 min (LY30), estimated percent lysis (EPL), and coagulation index (CI). Immune indicators included anti-nuclear antibody (ANA), anti-double stranded DNA antibody (dsDNA Ab), anti-SSA antibody (SSA Ab), anti-SSB antibody (SSB Ab), anti-Pm Scl antibody (Pm-Scl Ab), anti-cardiolipin antibody (ACA Ab), and standardized ratio of lupus anticoagulant dRVVT.

Information collected on the birth status of newborns included presence of thrombocytopenia at birth, preterm birth rate, neonatal asphyxia rate, admission to neonatal intensive care unit (NICU), low birth weight infants, 1-min and 5-min Apgar scores, and birth weight.

### 2.3 Statistical analysis

We used IBM SPSS Statistics v27.0 software for statistical analysis. Normal distribution continuous data are represented as mean ± SD. For multiple sample means, one-way analysis of variance was used. If there were significant inter group differences in the results, Tukey’s test was used for pairwise comparison; for the mean of two paired samples, paired *t*-test was used. If the data did not follow a normal distribution, we used the median (interquartile range) to represent it. For comparing multiple sets of samples, we used Kruskal Wallis test. If there was a significant difference in the results, we used Mann Whitney U test for pairwise comparison; for the comparison of two paired samples, Wilcoxon signed rank test was used. The categorical data is represented as (%) and analyzed using chi square test. For categorical data that did not meet the chi square test hypothesis, Fisher exact test was used. To correct type I errors in pairwise comparisons, Bonferroni correction was used. We used Pearson correlation analysis to calculate the relationship between TEG parameters and prenatal platelets. *P* < 0.05 was considered statistically significant.

## 3 Results

### 3.1 Distribution of causes and severity of thrombocytopenia in pregnancy

**FIGURE 1:**
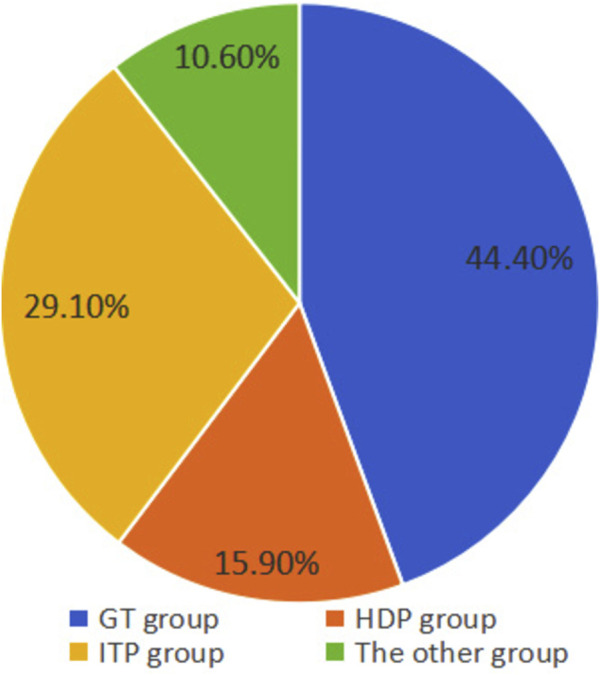
Distribution of causes in pregnant women with thrombocytopenia. The GT group has the largest proportion, followed by the ITP group, the HDP group, and the other group.

**FIGURE 2:**
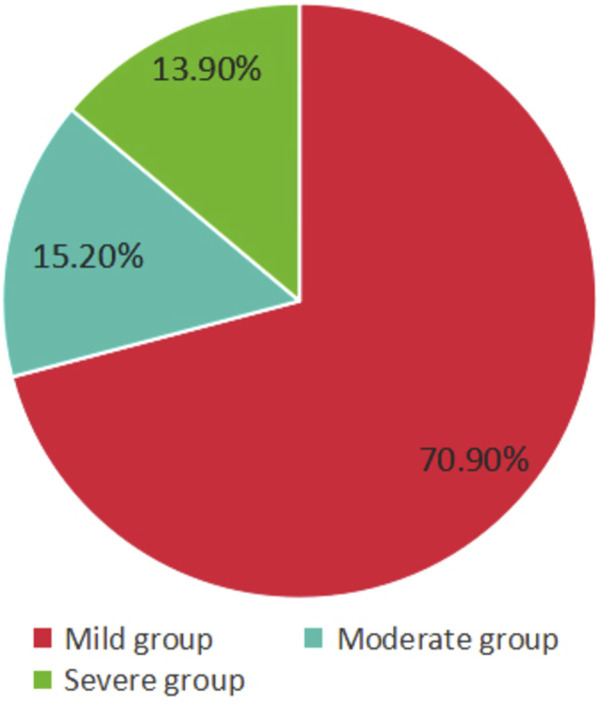
Severity distribution of pregnant women with thrombocytopenia. The mild group owns the highest proportion.

According to the etiology of thrombocytopenia, a total of 67 cases (44.40%) were in the GT group, 24 cases (15.90%) in the HDP group, 44 cases (29.10%) in the ITP group, and 16 cases (10.60%) in the other group (Figure 1). According to the severity of thrombocytopenia, there were 107 cases (70.90%) in the mild group, 23 cases (15.20%) in the moderate group, and 21 cases (13.90%) in the severe group (Figure 2).

### 3.2 Comparison of clinical characteristics

Grouping according to the etiology of thrombocytopenia, pairwise comparisons between groups showed that patients in the ITP group were significantly younger than those in the GT group and the other group. The ITP group had a higher proportion of skin and mucosal bleeding during pregnancy (43.18%), the smallest gestational age at the first diagnosis, the lowest platelet count at the time of the first diagnosis, during pregnancy and on the third day postpartum. The lowest platelet count during pregnancy was ranked from low to high: ITP group < the other group < HDP group < GT group, and the difference was statistically significant (*P* < 0.05) (Table 1).

**TABLE 1:** Comparison of clinical characteristics of patients.

Groups	Age (years)	Skin and mucosa bleeding during pregnancy (%)	Gestational age at first diagnosis (week)	Platelet count at first diagnosis (×10^9^/L)	Lowest platelet count during pregnancy (×10^9^/L)	Platelet count on the third day after delivery (×10^9^/L)
Grouping according to etiology						
GT group (*n =* 67)	30.00 (27.00–34.00)	12 (17.91)	36.00 (31.00–39.00)	88.00 (80.00–93.50)	83.00 (76.50–89.50)	108.00 (91.00–121.00)
HDP group (*n =* 24)	29.00 (27.00–32.00)	4 (16.67)	35.00 (33.50–37.00)	78.50 (67.00–89.50)^1^	76.50 (57.50–81.00)^1^	108.00 (96.50–123.50)
ITP group (*n =* 44)	28.00 (26.00–31.00)^1^	19 (43.18)^1^	24.00 (12.00–34.00)^1^ ^,^ ^2^	53.00 (35.00–74.00)^1^ ^,^ ^2^	36.00 (22.50–50.00)^1^ ^,^ ^2^	66.00 (46.50–96.50)^1^ ^,^ ^2^
The other group (*n =* 16)	30.50 (28.50–33.00)^3^	2 (12.50)	24.00 (12.00–34.00)^1^	86.00 (50.50–92.50)^1^ ^,^ ^3^	56.50 (34.00–88.50)^3^	86.00 (61.00–106.00)^1^
*H*/χ^2^	10.89	11.92	35.05	57.32	76.11	30.00
*P*	0.012^*^	0.008^*^	<0.001^*^	<0.001^*^	<0.001^*^	<0.001^*^
Grouping according to severity						
Mild group (*n =* 107)	30.00 (28.00–34.00)	16 (14.95)	36.00 (31.00–39.00)	86.00 (76.00–93.00)	-	106.00 (91.00–119.00)
Moderate group (*n =* 23)	27.00 (25.50–30.00)^4^	9 (39.13)^4^	16.00 (12.00–28.00)^4^	59.00 (46.00–76.00)^4^	-	63.00 (51.00–82.00)^4^
Severe group (*n =* 21)	29.00 (27.00–29.00)	12 (57.14)^4^	24.00 (16.00–32.00)^4^	30.00 (21.00–41.50)^4^ ^,^ ^5^	-	47.00 (39.00–107.00)^4^
*H*/χ^2^	9.66	20.03	37.50	58.50	-	33.66
*P*	0.008^*^	<0.001^*^	<0.001^*^	<0.001^*^	-	<0.001^*^

*P < 0.05.

1 Compared with the GT, group.

2 Compared with the HDP, group.

3 Compared with the ITP, group.

4 Compared with the mild group.

5 Compared with the moderate group.

Grouping according to the severity of thrombocytopenia, pairwise comparisons between groups showed that the age of the mild group was significantly higher than that of the moderate group; the mild group had the highest platelet count at the first diagnosis of gestational age, and the highest platelet count on the third day after delivery, with statistical significance (*P* < 0.05) (Table 1).

### 3.3 Comparison of delivery outcomes and delivery methods

According to the etiological grouping, there was no statistically significant difference in the ICU transfer rate and postpartum hemorrhage between the groups. However, there were significant differences in the length of hospital stay, gestational age, and delivery mode. The pairwise comparison showed that the length of hospital stay in the GT group was significantly lower than those in the HDP and ITP groups. Patients in the GT group had the largest gestational age during delivery, and the proportion of cesarean section in the GT group was significantly higher than that in the HDP group (*P* < 0.05) (Table 2).

**TABLE 2:** Comparison of delivery outcomes and delivery modes of pregnant women.

Groups	ICU transfer (%)	Hospital length of stay (days)	Gestational age at delivery (week)	Postpartum hemorrhage (%)	Delivery mode (%)	
					Vaginal delivery	Cesarean section
Grouping according to etiology						
GT group (*n =* 67)	0 (0.00)	5.00 (4.00–6.00)	39.00 (39.00–40.00)	2 (3.00)	18 (26.90)	49 (73.10)
HDP group (*n =* 24)	2 (8.30)	7.00 (5.00–7.50)^1^	37.00 (35.00–38.50)^1^	1 (4.20)	0 (0.00)^1^	24 (100.00)^1^
ITP group (*n =* 44)	1 (2.30)	7.00 (4.50–8.00)^1^	38.00 (37.00–39.00)^1^	1 (2.30)	5 (11.40)	39 (88.60)
The other group (*n =* 16)	0 (0.00)	5.00 (4.00–8.50)	37.50 (37.00–39.00)^1^	0 (0.00)	1 (6.30)	15 (93.80)
*H*/Fisher	5.05	14.35	36.44	0.91	12.36	
*P*	0.063	0.002^*^	<0.001^*^	1.000	0.006^*^	
Grouping according to severity						
Mild group (*n =* 107)	1 (0.90)	5.00 (4.0–6.0)	39.00 (38.00–40.00)	3 (2.80)	21 (19.60)	86 (80.40)
Moderate group (*n =* 23)	1 (4.30)	7.00 (4.50–8.00)	37.00 (37.00–39.00)^2^	0 (0.00)	3 (13.00)	20 (87.00)
Severe group (*n =* 21)	1 (4.80)	7.00 (6.00–10.0)^2^	37.00 (35.00–39.00)^2^	1 (4.80)	0 (0.00)	21 (100.00)
*H*/Fisher	3.14	14.80	19.45	1.02	5.66	
*P*	0.204	<0.001^*^	<0.001^*^	0.533	0.055	

*P < 0.05.

1 Compared with the GT, group.

2 Compared with the mild group.

According to the severity of thrombocytopenia, there were no statistically significant differences in the ICU transfer rate, postpartum hemorrhage, and delivery mode among the groups. Comparison between groups showed that the hospitalization days of patients in the mild group were significantly lower than those in the severe group, and the mild group had the largest gestational age at delivery, with statistical significance (*P* < 0.05) (Table 2).

### 3.4 Comparison of the effectiveness of diverse treatments of thrombocytopenia

Among the 151 study subjects, 140 pregnant women received thrombocytopenia related treatments during pregnancy. According to the etiology, only patients in the ITP group were treated with IVIg. After treatments with glucocorticoids and glucocorticoids plus platelet transfusion, both the GT group and the HDP group patients showed a significant increase in platelet counts, indicating a significant therapeutic effect. The ITP group also achieved good therapeutic effects after treatments with glucocorticoids and platelet transfusion, and the difference was statistically significant (*P* < 0.05). However, the treatment of ITP with glucocorticoids alone was not effective, as shown in Table 3.

**TABLE 3:** Comparison of clinical effects of different treatments.

Groups	Number of effective cases (%)	Number of non-effective cases (%)	Platelet count before treatment (×10^9^/L)	Platelet count after treatment (×10^9^/L)	*t*	*P*
Grouping according to etiology						
GT group (*n =* 61)						
Glucocorticoids (*n =* 54)	36 (66.70)	18 (33.30)	89.33 ± 16.68	103.51 ± 20.54	5.60	<0.001^*^
Glucocorticoids + platelet transfusion (*n =* 7)	7 (100.00)	0 (0.00)	80.17 ± 9.33	107.50 ± 17.59	4.15	0.009^*^
Total	43	18				
HDP group (*n =* 23)						
Glucocorticoids (*n =* 13)	12 (72.30)	1 (7.70)	81.00 (77.00–94.00)	109.00 (94.50–121.00)	2.76^a^	0.006^*^
Platelet transfusion (*n =* 1)	1 (100.00)	0 (0.00)	-	-	-	-
Glucocorticoids + platelet transfusion (*n =* 9)	7 (79.80)	2 (22.80)	58.00 ± 29.00	79.13 ± 44.78	2.41	0.047^*^
Total	20	3				
ITP group (*n =* 42)						
Glucocorticoids (*n =* 14)	8 (57.10)	6 (42.90)	62.50 (57.00–91.00)	70.00 (57.00–92.00)	1.08^a^	0.279
IVIg (*n =* 1)	1 (100.00)	0 (0.00)	-	-	-	-
Platelet transfusion (*n =* 7)	6 (85.70)	1 (14.30)	375.43 ± 10.50	51.71 ± 11.66	2.06	0.086
Glucocorticoids + platelet transfusion (*n =* 20)	17 (85.00)	3 (15.00)	35.50 (21.00–56.50)	65.00 (45.50–95.00)	3.87^a^	<0.001^*^
Total	32	10				
The other group (*n =* 14)						
Glucocorticoids (*n =* 10)	4 (40.00)	6 (60.00)	78.30 ± 23.74	81.20 ± 17.94	0.43	0.678
Platelet transfusion (*n =* 1)	1 (100.00)	0 (0.00)	-	-	-	0.288
Glucocorticoids + platelet transfusion (*n =* 3)	3 (100.00)	0 (0.00)	43.00 (37.00–57.50)	112.00 (83.50–16.50)	1.60^a^	0.109
Total	8	6				
Grouping according to severity						
Mild group (*n =* 98)						
Glucocorticoids (*n =* 79)	52 (65.80)	27 (34.20)	88.01 ± 16.87	101.89 ± 23.44	5.98	<0.001^*^
Platelet transfusion (*n =* 1)	1 (100.00)	0 (0.00)	-	-	-	-
Glucocorticoids + platelet transfusion (*n =* 18)	17 (94.40)	1 (5.60)	75.00 (66.00–82.00)	103.00 (93.00–114.00)	3.68^a^	<0.001^*^
Total	70	28				
Moderate group (*n =* 21)						
Glucocorticoids (*n =* 9)	5 (55.60)	4 (44.40)	61.00 ± 21.47	66.78 ± 17.33	0.77	0.461
Platelet transfusion (*n =* 4)	4 (100.00)	0 (0.00)	37.00 (33.00–42.50)	51.00 (47.00–58.50)	1.83^a^	0.068
Glucocorticoids + platelet transfusion (*n =* 8)	7 (87.50)	1 (12.50)	42.50 (39.00–53.00)	67.00 (53.50–87.50)	2.52^a^	0.012^*^
Total	16	5				
Severe group (*n =* 21)						
Glucocorticoids (*n =* 3)	3 (100.00)	0 (0.00)	47.00 (43.50–72.50)	135.00 (97.00–135.50)	1.60^a^	0.109
IVIg (*n =* 1)	1 (100.00)	0 (0.00)	-	-		-
Platelet transfusion (*n =* 4)	3 (75.00)	1 (25.00)	30.00 (24.00–39.50)	40.50 (37.00–59.5)	1.10^a^	0.273
Glucocorticoids + platelet transfusion (*n =* 13)	10 (76.90)	3 (23.10)	21.00 (19.00–24.00)	46.00 (39.00–67.00)	2.97^a^	0.003^*^
Total	17	4				

*P < 0.05.

^a^ Nonparametric Wilcoxon signed rank test was used.

According to the severity of thrombocytopenia, only patients in the severe group were treated with IVIg. In comparison among the groups, the mild group received significant effects of glucocorticoid or glucocorticoid plus platelet transfusion therapy. After treatments with glucocorticoids plus platelet transfusion, the platelet count significantly increased in the moderate and severe groups, and the therapeutic effect was statistically significant (*P* < 0.05). However, for patients in the moderate and severe groups, the use of glucocorticoids alone or platelet transfusion alone did not achieve satisfactory therapeutic effects (Table 3).

### 3.5 Comparison of laboratory indexes

#### 3.5.1 Comparison of blood routine and coagulation function

Grouped by etiology, postpartum platelet counts and Fg levels in each group were significantly higher than those before delivery. In the ITP group and the other group, postpartum PT and INR values were significantly higher than their prenatal values. For the GT group and the ITP group, APTT values after delivery were significantly higher than those before delivery. The postpartum TT values in the other group were significantly lower compared to the prenatal numbers (*P* < 0.05). In addition, postpartum Hb concentrations in the GT group, ITP group, and the other group were significantly lower than those before delivery. Postpartum PDW and MPV counts were significantly lower than the prenatal counts before delivery among the three groups. Postpartum PCT counts in the GT group, HDP group and ITP group were significantly higher than the prenatal counts. In the GT group and ITP group, postpartum P-LCR values were significantly lower than the prenatal values (Table 4).

**TABLE 4:** Comparison of blood routine and coagulation function in the first week before delivery and the third day after delivery.

Grouping according to etiology		
Groups	Prenatal count	Postnatal count
	Platelet	
GT group	89.00 (80.00–97.00)	108.00 (91.00–121.00)^1^
HDP group	80.00 (66.00–87.50)	108.00 ^(96.50–123.50)^ ^1^
ITP group	46.50 (31.00–64.00)	66.00 (46.50–96.50)^1^
The other group	69.00 (44.50–90.00)	86.00 (61.00–106.00)^1^
*P*	<0.001^*^	<0.001^*^
Hb		
GT group	119.00 (111.00–126.50)	106.52 ± 18.48^1^
HDP group	114.50 (107.00–123.00)	104.00 ± 20.43
ITP group	107.00 (95.00–118.50)	98.23 ± 17.32^1^
The other group	120.00 (107.50–128.50)	104.13 ± 14.64^1^
*P*	0.006^*^	0.138
PDW		
GT group	17.06 ± 3.73	15.30 (12.70–18.70)^1^
HDP group	17.12 ± 3.77	14.95 (13.30–17.20)^1^
ITP group	16.00 ± 4.00	13.50 (11.60–14.90)^1^
The other group	14.86 ± 2.06	14.00 (11.60–15.65)^1^
*P*	0.16	0.042^*^
MPV		
GT group	12.35 ± 1.20	11.90 (11.10–13.05)^1^
HDP group	12.41 ± 1.04	11.80 (10.90–12.50)^1^
ITP group	12.20 ± 1.48	11.30 (10.60–12.00)^1^
The other group	11.94 ± 0.93	11.30 (10.65–11.80)^1^
*P*	0.679	0.041^*^
PCT		
GT group	0.10 (0.10–0.12)	0.10 (0.10–0.15)^1^
HDP group	0.10 (0.08–0.10)	0.10 (0.10–0.14)^1^
ITP group	0.06 (0.00–0.10)	0.10 (0.08–0.10)^1^
The other group	0.09 (0.07–0.10)	0.10 (0.10–0.10)
*P*	<0.001^*^	<0.001^*^
P-LCR		
GT group	41.20 (33.10–50.65)	39.30 (33.15–46.50)^1^
HDP group	39.65 (28.75–49.40)	40.20 (31.80–41.80)
ITP group	38.50 (24.90–47.50)	34.00 (27.10–39.20)^1^
The other group	37.70 (32.40–43.55)	34.95 (29.20–39.20)
*P*	0.374	0.025^*^
PT		
GT group	12.40 (12.00–12.90)	12.65 (12.10–12.90)
HDP group	12.10 (11.70–12.60)	12.30 (11.40–12.50)
ITP group	12.50 (12.10–12.90)	12.90 (12.30–13.20)^1^
The other group	11.95 (11.70–12.20)	12.50 (11.95–12.85)^1^
*P*	0.294	0.012^*^
APTT		
GT group	32.68 ± 2.34	33.64 ± 3.52^1^
HDP group	33.58 ± 4.05	33.28 ± 5.33
ITP group	32.66 ± 2.76	34.22 ± 3.48^1^
The other group	32.22 ± 3.29	33.25 ± 4.08
*P*	0.475	0.837
D-dimer		
GT group	1.44 (1.17–2.13)	1.36 (1.00–1.82)
HDP group	1.68 (0.89–2.55)	1.42 (0.86–2.20)
ITP group	1.85 (1.07–2.55)	1.69 (1.21–2.45)
The other group	1.34 (0.95–1.87)	1.29 (0.96–1.75)
*P*	0.36	0.125
INR		
GT group	1.00 (0.90–1.00)	0.97 (0.93–1.00)
HDP group	1.00 (0.94–1.00)	1.00 (0.96–1.00)
ITP group	1.00 (0.96–1.00)	1.00 (1.00–1.00)^1^
The other group	0.99 (0.89–1.00)	1.00 (0.93–1.00)^1^
*P*	0.182	0.058
TT		
GT group	15.50 (14.75–16.00)	15.40 (15.00–16.00)
HDP group	16^.40 (15.85–17.20)^	15.95 (15.55–16.50)
ITP group	15.50 (15.20–15.85)	15.55 (14.80–15.90)
The other group	15.70 (15.35–16.45)	15.40 (14.70–15.80)^1^
*P*	0.004^*^	0.165
Fg		
GT group	4.35 (3.84–4.83)	4.87 (3.83–5.35)^1^
HDP group	4.17 (3.30–4.47)	4.76 (4.11–5.15)^1^
ITP group	4.18 (3.61–5.00)	4.48 (4.08–5.11)^1^

Grouping according to severity		
The other group	4.42 (3.86–4.91)	5.03 (4.43–5.77)^1^
*P*	0.373	0.472
Platelet		
Mild group	87.00 (73.00–94.00)	106.00 (91.00–119.00)^1^
Moderate group	46.00 (39.50–61.00)	63.00 (51.00–82.00)^1^
Severe group	26.00 (20.00–40.00)	47.00 (39.00–107.00)^1^ ^)^
*P*	<0.001^*^	<0.001^*^
Hb		
Mild group	118.00 (109.00–126.00)	105.94 ± 18.19^1^ ^)^
Moderate group	111.00 (104.50–124.00)	101.70 ± 15.33^1^
Severe group	103.00 (86.50–110.50)	92.05 ± 17.93
*P*	<0.001^*^	0.006^*^
PDW		
Mild group	15.80 (14.40–20.00)	15.00 (12.90–18.40)^1^
Moderate group	16.20 (14.50–16.90)	13.75 (12.80–15.40)^1^
Severe group	14.65 (12.15–17.00)	12.10 (11.10–14.30)^1^
*P*	0.252	0.001^*^
MPV		
Mild group	12.32 ± 1.20	11.80 (11.05–12.85)^1^
Moderate group	12.15 ± 1.30	11.55 (10.90–12.00)^1^
Severe group	12.08 ± 1.35	10.95 (10.10–11.50)^1^
*P*	0.772	0.003^*^
Moderate group	15.50 (14.90–16.00)	15.39 ± 0.66
Severe group	15.50 (15.30–15.70)	15.09 ± 0.75
*P*	0.569	0.085
Fg		
Mild group	4.29 (3.80–4.87)	4.71 ± 0.93^1^
Moderate group	4.14 (3.51–4.82)	4.61 ± 0.50
Severe group	4.20 (3.78–4.93)	4.66 ± 1.12^1^
*P*	0.727	0.905

*P < 0.05.

1 Comparison with the same index before delivery.

According to the severity of thrombocytopenia, postpartum platelet and PCT counts in each group were significantly higher than the prenatal counts. The postpartum PT value in the severe group was significantly higher compared to the prenatal value. In the mild and moderate groups, APTT values after delivery were significantly higher than those of before delivery. In addition, the postpartum D-dimer value of the mild group was significantly lower than that of the prenatal value. Postpartum Fg levels in the mild group and the severe group were significantly higher compared to the prenatal levels. The postpartum Hb counts and P-LCR values in the mild group and the moderate group were significantly lower than those before delivery. The postpartum PDW and MPV counts of each group were significantly lower than the prenatal counts (*P* < 0.05) (Table 4).

#### 3.5.2 Correlation between platelet count and TEG parameters

We collected platelet count and TEG parameters which were both tested in our hospital within 1 week before delivery. R value, angle, Ma value and CI value were positively correlated with platelet count in the other group, while K value was negatively correlated with platelet count in the other group. There was no correlation between platelet count and TEG parameters in the GT group, HDP group and ITP group (Table 5).

**TABLE 5:** The correlation between prenatal platelet count and TEG parameters.

Indicators	GT group (*n =* 39)		HDP group (*n =* 13)		ITP group (*n =* 13)		The other group (*n =* 12)	
	r	*P*	r	*P*	r	*P*	r	*P*
Grouping according to etiology								
R (mins)	−0.47	0.207	−0.26	0.830	−0.73	0.479	1.00	
K (mins)	−0.39	0.307	−0.99	0.099	−0.92	0.252	−1.00	
Angle (º)	0.04	0.928	0.96	0.180	0.89	0.304	1.00	
MA (mm)	0.42	0.262	0.83	0.377	0.99	0.085	1.00	
LY30 (%)	−0.19	0.744	0.00	1.000	N/A	N/A	N/A	N/A
EPL (%)	−0.13	0.744	0.00	1.000	N/A	N/A	N/A	N/A
CI	0.20	0.600	0.95	0.200	0.90	0.282	1.00	

Indicators	Mild group (*n =* 53)		Moderate group (*n =* 12)		Severe group (*n =* 12)	
	r	*P*	r	*P*	r	*P*
Grouping according to severity						
R (mins)	−0.27	0.365	−1.00		−1.00	
K (mins)	−0.54	0.058	−1.00		−1.00	
Angle (º)	0.55	0.051	1.00		1.00	
MA (mm)	0.44	0.137	1.00		1.00	
LY30 (%)	−0.19	0.533	N/A	N/A	N/A	N/A
EPL (%)	−0.19	0.533	N/A	N/A	N/A	N/A
CI	0.55	0.054	1.00		1.00	

Note: “.” due to the existence of complete correlation, significance was not calculated; N/A, not applicable.

Grouped by severity, R value and K value were negatively correlated with the platelet count in the moderate and severe groups, while angle, Ma value and CI value were positively correlated with the platelet count in the moderate and severe groups. TEG parameters were not correlated with the platelet count in the mild group (Table 5).

#### 3.5.3 Comparison of immune indexes

There was no significant difference in the immune indexes of ANA, dsDNA Ab, SSA Ab, SSB Ab, Pm-Scl Ab, ACA Ab and dRVVT among all groups, regardless of grouping based on the etiology or the severity of thrombocytopenia (Table 6).

**TABLE 6:** Comparison of positive immune antibodies of pregnant women.

Indicators	GT group (%) (*n =* 16)	HDP group (%) (*n =* 8)	ITP group (%) (*n =* 14)	The other group (%) (*n =* 10)	*P*
Grouping according to etiology					
ANA>1:80	2 (12.50)	0 (0.00)	0 (0.00)	2 (20.00)	0.262
dsDNA Ab	0 (0.00)	0 (0.00)	0 (0.00)	1 (10.00)	0.375
SSA Ab	0 (0.00)	0 (0.00)	1 (7.69)	0 (0.00)	0.667
SSB Ab	0 (0.00)	0 (0.00)	0 (0.00)	0 (0.00)	N/A
Pm-Scl Ab	0 (0.00)	1 (12.50)	0 (0.00)	0 (0.00)	0.167
ACA Ab	0 (0.00)	0 (0.00)	0 (0.00)	1 (10.00)	0.658
dRVVT>1.20	0 (0.00)	0 (0.00)	0 (0.00)	1 (10.00)	0.286

Indicators	Mild group (%) (*n =* 28)	Moderate group (%) (*n =* 9)	Severe group (%) (*n =* 11)	*P*
Grouping according to severity				
ANA>1:80	3 (10.71)	1 (11.11)	0 (0.00)	0.622
dsDNA Ab	0 (0.00)	0 (0.00)	1 (9.09)	0.417
SSA Ab	0 (0.00)	1 (11.11)	0 (0.00)	0.208
SSB Ab	0 (0.00)	0 (0.00)	0 (0.00)	N/A
Pm-Scl Ab	1 (3.57)	0 (0.00)	0 (0.00)	1.000
ACA Ab	0 (0.00)	0 (0.00)	1 (9.09)	0.421
dRVVT>1.20	0 (0.00)	1 (11.11)	0 (0.00)	0.143

Note: Fisher test was used; N/A, not applicable.

### 3.6 Comparison of newborn birth conditions

Grouping based on the etiology of thrombocytopenia, there were no significant differences in the rate of thrombocytopenia at birth, the rate of premature birth, the rate of asphyxia, and the Apgar scores at 1 min and 5 min among the groups. In pairwise comparison, the NICU transfer rate and the incidence of low birth weight infants in the HDP group were significantly higher than those in the GT group, and the neonatal birth weight in the HDP group was significantly lower than those in the GT group and the ITP group (*P* < 0.05) (Table 7).

**TABLE 7:** Comparison of birth conditions of newborns.

Groups	Thrombocytopenia at birth (%)	Preterm birth rate (%)		Asphyxia rate (%)	NICU transfer (%)	Low birth weight (%)	Apgar score		Weight (grams)
		<34 weeks	>34 weeks				1 min	5 min	
Grouping according to etiology									
GT group (*n =* 67)	1 (5.30)	1 (25.00)	3 (75.00)	1 (1.50)	5 (7.50)	1 (1.50)	10.00 (10.00–10.00)	10.00 (10.00–10.00)	3335.00 (3140.00–3660.00)
HDP group (*n =* 24)	0 (0.00)	2 (28.60)	5 (71.40)	0 (0.00)	8 (33.30)^1^	9 (37.50)^1^	10.00 (9.50–10.00)	10.00 (10.00–10.00)	2720.00 (2210.00–3125.00)^1^
ITP group (*n =* 44)	2 (20.00)	7 (77.70)	2 (22.20)	1 (2.30)	7 (15.90)	5 (11.40)	10.00 (10.00–10.00)	10.00 (10.00–10.00)	3165.00 (2740.00–3535.00)^2^
The other group (*n =* 16)	1 (25.00)	3 (100.00)	0 (0.00)	0 (0.00)	4 (25.00)	3 (18.80)	10.00 (10.00–10.00)	10.00 (10.00–10.00)	3135.00 (2690.00–3520.00)
*H*/Fisher	3.11	7.07		1.27	9.88	21.02	5.42	4.92	25.20
*P*	0.276	0.071		1.000	0.015^*^	<0.001^*^	0.144	0.178	<0.001^*^
Grouping according to severity									
Mild group (*n =* 107)	3 (10.00)	3 (33.30)	6 (66.70)	1 (0.90)	14 (13.10)	8 (7.50)	10.00 (10.00–10.00)	10.00 (10.00–10.00)	3330.00 (2995.00–3565.00)
Moderate group (*n =* 23)	1 (16.70)	4 (66.70)	2 (33.30)	0 (0.00)	4 (17.40)	2 (8.70)	10.00 (8.50–10.00)^3^	10.00 (10.00–10.00)	3110.00 (2820.00–3250.00)
Severe group (*n =* 21)	0 (0.00)	6 (75.00)	2 (25.00)	1 (4.80)	6 (28.60)	8 (38.10)^3^	10.00 (10.00–10.00)	10.00 (10.00–10.00)	2580.00 (2390.00–3160.00)^3^
*H*/Fisher	1.02	3.16		2.40	3.25	12.18	7.03	0.45	14.04
*P*	0.629	0.183		0.282	0.188	<0.001^*^	0.030^*^	0.800	<0.001^*^

*P < 0.05.

1 Compared with the GT, group.

2 Compared with the HDP, group.

3 Compared with the mild group.

Grouping according to the severity of thrombocytopenia, there were no significant differences in the rates of neonatal thrombocytopenia, premature birth, asphyxia, NICU transfer, and 5-min Apgar score among the groups. The 1-min Apgar score of newborns in the mild group was significantly higher than that in the moderate group, and the birth weight of newborns in the severe group was significantly lower than that in the mild group (Table 7).

## 4 Discussion

Thrombocytopenia in pregnancy can cause serious adverse consequences, leading to postpartum hemorrhage, hemorrhagic shock, and neonatal intracranial hemorrhage. In this study, GT group accounted for the largest proportion, followed by the ITP group, the HDP group and the other group. Grouped by the severity of thrombocytopenia, 70.90% of patients belong to the mild group, 15.20% in the moderate group, and only 13.90% in the severe group. In terms of clinical manifestations, the ITP group had a higher proportion of skin and mucous membrane bleeding during pregnancy (43.18%), the smallest gestational age at first diagnosis and the lowest platelet count at first diagnosis. In terms of delivery outcomes, the length of hospital stay in the GT group was significantly lower than that in the HDP group and the ITP group, and the gestational age of delivery was the largest, suggesting that the condition of GT group might be mild. Regarding the treatments of thrombocytopenia in pregnancy, the platelet counts of the GT group and the HDP group increased significantly after the treatment of glucocorticoid alone or glucocorticoid plus platelet infusion. However, the effect of glucocorticoid alone in the ITP group was not good, thus the combination therapy was needed to achieve better effect. In addition, postpartum Hb counts in the GT group, the ITP group and the other group were significantly lower than those in prenatal, suggesting that thrombocytopenia could aggravate the loss of Hb for these patients. TEG parameters were correlated with the prenatal platelet count of patients in the moderate and the severe groups, but not with the mild group, indicating that only patients in the moderate and severe groups could cause changes in blood coagulation and fibrinolysis system. For the newborns, the NICU transfer rate and the incidence of low birth weight infants in the HDP group were significantly higher than those in the GT group, and the birth weight of newborns delivered by pregnant women in the HDP group was lower, indicating that HDP had a greater impact on newborns, which needed special attention.

As for the etiology of thrombocytopenia in pregnancy, our results are slightly different from other reports (Parnas et al., 2006; Yuce et al., 2014). They believed that the incidence of HDP was higher than that of ITP, but we found that the incidence of HDP was lower than that of ITP. The difference may be related to the different research subjects. The unified viewpoint is that GT is still the most common cause of thrombocytopenia in pregnancy (Yan et al., 2016; Park, 2022; Fogerty, 2018). In the present study, 70.90% of cases belong to the mild group, which is consistent with other studies (Gernsheimer et al., 2013; Govindappagari et al., 2020).

In this paper, the platelet count of ITP group during pregnancy and the third day after delivery is the lowest, which is consistent with the nature of ITP. ITP is a disease caused by immune-mediated platelet destruction (Fogerty, 2018) or decreased platelet production (Fogerty, 2024), which is usually associated with more severe thrombocytopenia and bleeding. The research of Rodeghiero et al. (2009) provided a comprehensive analysis for the classification and severity of ITP, emphasizing that the increased risk of bleeding is the main clinical problem of ITP, which depends on the degree of thrombocytopenia. In addition, the number of gestational weeks at the first diagnosis of ITP group was the lowest, which showed that compared with other groups, ITP often appeared in the early pregnancy. This is of great clinical significance because it emphasizes the necessity of early screening and management of suspected ITP cases. In contrast to ITP group, the GT group had fewer symptoms of skin mucosal bleeding during pregnancy, and maintained higher platelet counts in pregnancy and postpartum. GT may be due to hemodilution caused by the increase of plasma volume during pregnancy (Pishko et al., 2020), which is different from ITP. Additionally, the hospital stay of patients in the GT group was shorter than that in the HDP group and the ITP group, which showed that although thrombocytopenia existed, the complexity of GT was relatively low. Moreover, the gestational week of delivery in the GT group was higher, suggesting that GT did not seem to increase the risk of adverse pregnancy outcomes (Cines and Levine, 2017a).

Regarding the mode of delivery, women undergoing elective cesarean section have been associated with an increased risk of blood loss and blood transfusion (Attali et al., 2021), hence, the indications of cesarean section should be determined according to the obstetric situation. Researchers believe that cesarean section is safe and feasible when the platelet count reaches is more than 50 × 10^9^/L (Provan et al., 2019). At present, it is generally believed that cesarean section can be considered for full-term pregnancy with platelet count less than 50 × 10^9^/L and bleeding tendency (Myers, 2012). For full-term pregnancy with platelet count more than 50 × 10^9^/L, if there is no indication of obstetric cesarean section, vaginal natural delivery can be considered (Myers, 2012). The reason for the increase of cesarean section rate in the GT group in our study may be due to the patients’ fear of fetal intracranial hemorrhage. In the future, it is necessary to encourage these mild GT patients to have vaginal delivery if the condition allows.

In the treatment of thrombocytopenia, the efficacy of platelet transfusion has been fully affirmed (Kaufman et al., 2015), and the transfusion of a therapeutic amount of platelet will increase the platelet count by about 5–10 × 10^9^/L (Bauer et al., 2021). However, for massive hemorrhage (platelet count <10 × 10^9^/L), platelet transfusion has no significant effect on reducing mortality (Stanworth and Shah, 2022). Platelet transfusion is suitable for patients with impaired platelet formation or increased platelet destruction, but platelet transfusion may be harmful to patients with increased intravascular platelet activation (Greinacher and Selleng, 2016). At present, general supportive care with a combination of treatments, including corticosteroids, IVIg, and platelet transfusion has been recommended for a more effective and rapid increase of platelet count for treatment of life-threatening hemorrhage due to ITP, and in the absence of significant response, the early addition of a thrombopoietin receptor agonists (TPO-Ras) should also be considered (Provan et al., 2019). A recent systematic review suggested that pregnant women with ITP might be suitable for TPO-Ras treatment, although it was off-label (Snow et al., 2023). In our study, patients in the ITP group benefited from the combined treatment of glucocorticoid and platelet transfusion. If only glucocorticoid was used, the effect was not good. It has been reported that patients with moderate and severe thrombocytopenia should be treated with glucocorticoid and platelet transfusion before cesarean section, in order to quickly stabilize the platelet level and reduce intraoperative bleeding (Gernsheimer et al., 2013). The American Society of Hematology (ASH) 2019 guidelines also recommend that pregnant women with ITP receive corticosteroids or IVIg, and the mode of delivery should be determined based on obstetric indications (Neunert et al., 2019). Moreover, we found that the platelet counts in the GT group and the HDP group treated with glucocorticoids alone increased significantly after treatment, which was consistent with another report (Woudstra et al., 2010). This indicates that monotherapy may be enough to treat mild thrombocytopenia.

Postpartum platelet counts in each group of patients are higher than those before delivery, which reflects the recovery of postpartum platelet production, because the physiological demand for platelet after delivery is reduced. For patients with thrombocytopenia in pregnancy, platelet consumption decreases after delivery, resulting in a significant increase in postpartum platelet count (Ushida et al., 2021). Moreover, PCT refers to the volume percentage of platelets in the blood (Budak et al., 2016). Monitoring PCT can help track the body’s response to platelet turnover/production, especially in severe cases. If PCT is reduced, a higher level of platelet production may be required to make up for the reduced platelets. The postpartum PCT level of patients in each group (except the other group) was significantly higher than that in prenatal, representing the recovery of postpartum platelet consumption. In addition, PDW is a marker of platelet size variability (Budak et al., 2016), and can predict coagulation activation (Liu et al., 2019). Therefore, the decrease of PDW may indicate stable platelet formation and turnover. MPV is a marker of platelet activation (Budak et al., 2016) and can also be considered an indicator of platelet function (Vizioli et al., 2009). The increase of MPV reflects the increase of platelet clearance or destruction (Cines and Levine, 2017a). The decrease of postpartum MPV in this study may represent the improvement of postpartum platelet count. Researchers proposed that MPV can be used to discriminate ITP from thrombocytopenia caused by decreased platelet production (i.e., hypo-productive thrombocytopenia) (Walle et al., 2023). Large platelets are mostly young platelets, and P-LCR refers to the presence of large platelets in the blood and is used to monitor platelet activity (Budak et al., 2016). In our study, the postpartum P-LCR levels in the GT group and the ITP group decreased significantly. Since the overall change trend of platelet count showed an increase after delivery, the decrease of postpartum P-LCR might indicate that platelet production was gradually recovering, and platelets were more mature and smaller in size.

Xie et al. (2021) established the reference interval of TEG parameters including R, K, Ma and α-angle of healthy pregnant women in the third trimester of pregnancy. They found that compared with normal women without pregnancy, R value decreased (without statistical significance), Ma increased significantly, which was consistent with the hypercoagulable state during pregnancy. TEG can help to detect and quantify platelet function, and it is useful to assist physicians in providing targeted medical interventions earlier (Dias et al., 2020). In general, a prolonged R value indicates a deficiency of coagulation factors, a prolonged K value shows a deficiency of fibrinogen, a decrease in MA value indicates either a reduction in platelets or abnormal platelet function, and a decreased CI value indicates a decrease in coagulation factors, a decrease in platelets, or an overactivity of the fibrinolytic system. The correlation between prenatal platelet count and TEG parameters in the HDP group was not significant, which indicated that for HDP patients, in addition to platelet count, there might be other factors that affect the coagulation process (Andersson et al., 2024; Davies et al., 2007; Spiezia et al., 2015). However, platelet counts in the moderate group and severe group were negatively correlated with R value and K value, indicating that lower platelet count resulted in delayed clot formation and weakened coagulation strength. Therefore, it is necessary to perform the TEG test for patients with moderate and severe thrombocytopenia, as these patients have a higher likelihood of blood transfusions. Changes in the R and K values can assist obstetricians in determining the type of blood product that needs to be transfused. Moreover, the traditional view is that cesarean section will aggravate the postpartum hypercoagulable state, because the R, K and α-angle after cesarean section are significantly shortened (Boyce et al., 2011). However, a few studies have revealed that the TEG parameters before and immediately after cesarean section are similar (Macafee et al., 2012; Sharma and Philip, 1997), which does not support the above view. A prospective study measured the changes of TEG parameters during the postnatal period up to 6 weeks after delivery and found that there was still a hypercoagulable state in the maternal body within 3 weeks after delivery (Saha et al., 2009). Compared with vaginal delivery, the thrombus parameters after cesarean section did not increase significantly (Saha et al., 2009). We did not test TEG after delivery, therefore, we were unable to analyze the relationship between postpartum platelet count and TEG parameters.

In clinical, thrombocytopenia not only occurs in ITP, but also in some secondary autoimmune diseases, such as systemic lupus erythematosus, APS, Sjögren’s syndrome, or rheumatoid arthritis. Therefore, we analyzed whether there was a correlation between immune indicators and the types of thrombocytopenia. In our study, there was no statistical difference in the autoimmune antibodies of patients in each group. It seems that immunity has no effect on thrombocytopenia in pregnancy. However, the number of patients undergoing autoimmune antibody testing is relatively small, thus this conclusion needs to be further confirmed by expanding the sample size.

We found that the incidence of low birth weight infants was the highest in the HDP group. This may be due to the impaired placentation caused by HDP, which increases the likelihood of fetal growth restriction (Di Martino et al., 2022). Similarly, the birth weight of newborns in the GT group was the highest, which was significantly higher than that in the HDP group and the ITP group. This is consistent with the mild systemic effects of GT, which is usually transient and self-limiting, and may have the least impact on fetal growth. Grouping according to the severity of thrombocytopenia, the incidence of low birth weight was higher in the severe group compared with the mild group, indicating that severe thrombocytopenia may affect the growth and development of newborns.

This study has some limitations. First, the sample size is limited, and larger sample size research is needed to further verify our results. Second, there may be a potential selection bias in our study. Third, we did not conduct long-term follow-up on the prognosis of newborns. In the future, we can collaborate with pediatricians to perform follow-up on neonates for a longer period, so as to provide more information for clinical practice.

## 5 Conclusion

In conclusion, ITP is associated with more severe thrombocytopenia and bleeding, often presenting in the early stage of pregnancy. Therefore, early screening and management should be carried out for suspected ITP cases. In the treatment of ITP, the combined use of glucocorticoids and platelet transfusion is recommended. GT patients have relatively mild clinical symptoms and less clinical harm, and do not seem to increase the risk of adverse pregnancy outcomes. For most patients with thrombocytopenia during pregnancy, postpartum Hb is significantly lower than that before delivery, suggesting that thrombocytopenia may have aggravated the loss of Hb in these patients. Obstetricians need to pay more attention to the prevention and treatment of postpartum hemorrhage. TEG parameter analysis suggests that patients in the moderate and severe groups may have changes in the blood coagulation and fibrinolysis systems, and changes in the coagulation function of these patients need to be monitored. Newborns delivered by HDP patients are more likely to be transferred to the NICU, and the probability of delivering low birth weight infants is increased. Therefore, more attention should be paid to the monitoring of these newborns. Our study provides new insights into the pregnancy outcomes of pregnant women with thrombocytopenia and lays a foundation for the development of targeted treatment strategies for these patients.

## Data Availability

The raw data supporting the conclusions of this article will be made available by the authors, without undue reservation.

## References

[B1] Andersson M. Bengtsson P. Karlsson O. Thörn S. E. Thorgeirsdottir L. Bergman L. (2024). Platelet aggregation and thromboelastometry monitoring in women with preeclampsia: a prospective observational study. Int. J. Obstet. Anesth. 61, 104297. 10.1016/j.ijoa.2024.104297 39837226

[B2] Attali E. Epstein D. Reicher L. Lavie M. Yogev Y. Hiersch L. (2021). Mild thrombocytopenia prior to elective cesarean section is an independent risk factor for blood transfusion. Arch. Gynecol. Obstet. 304 (3), 627–632. 10.1007/s00404-021-05988-x 33550466

[B3] Bar A. Moran R. Mendelsohn-Cohen N. Korem Kohanim Y. Mayo A. Toledano Y. (2025). Pregnancy and postpartum dynamics revealed by millions of lab tests. Sci. Adv. 11 (13), eadr7922. 10.1126/sciadv.adr7922 40138427 PMC11939066

[B4] Bauer M. E. Arendt K. Beilin Y. Gernsheimer T. Perez Botero J. James A. H. (2021). The society for obstetric Anesthesia and perinatology interdisciplinary consensus statement on neuraxial procedures in obstetric patients with thrombocytopenia. Anesth. Analg. 132 (6), 1531–1544. 10.1213/ANE.0000000000005355 33861047 PMC13109829

[B5] Boyce H. Hume-Smith H. Ng J. Columb M. O. Stocks G. M. (2011). Use of thromboelastography to guide thromboprophylaxis after caesarean section. Int. J. Obstet. Anesth. 20 (3), 213–218. 10.1016/j.ijoa.2011.03.006 21641791

[B6] Brown M. A. Magee L. A. Kenny L. C. Karumanchi S. A. McCarthy F. P. Saito S. (2018). Hypertensive disorders of pregnancy: ISSHP classification, diagnosis, and management recommendations for international practice. Hypertension 72 (1), 24–43. 10.1161/HYPERTENSIONAHA.117.10803 29899139

[B7] Budak Y. U. Polat M. Huysal K. (2016). The use of platelet indices, plateletcrit, mean platelet volume and platelet distribution width in emergency non-traumatic abdominal surgery: a systematic review. Biochem. Med. Zagreb. 26 (2), 178–193. 10.11613/BM.2016.020 27346963 PMC4910273

[B8] Cervera R. Tektonidou M. G. Espinosa G. Cabral A. R. González E. B. Erkan D. (2011). Task Force on Catastrophic Antiphospholipid Syndrome (APS) and Non-criteria APS Manifestations (II): thrombocytopenia and skin manifestations. Lupus. 20 (2), 174–181. 10.1177/0961203310395052 21303834

[B9] Cines D. B. Levine L. D. (2017a). Thrombocytopenia in pregnancy. Blood 130 (21), 2271–2277. 10.1182/blood-2017-05-781971 28637667 PMC5701522

[B10] Cines D. B. Levine L. D. (2017b). Thrombocytopenia in pregnancy. Hematol. Am. Soc. Hematol. Educ. Program 2017 (1), 144–151. 10.1182/asheducation-2017.1.144 29222249 PMC6142617

[B11] Connors J. M. Fein S. (2023). How to manage ITP with life-threatening bleeding. Hematol. Am. Soc. Hematol. Educ. Program 2023 (1), 254–258. 10.1182/hematology.2023000478 38066888 PMC10727002

[B12] Davies J. R. Fernando R. Hallworth S. P. (2007). Hemostatic function in healthy pregnant and preeclamptic women: an assessment using the platelet function analyzer (PFA-100) and thromboelastograph. Anesth. Analg. 104 (2), 416–420. 10.1213/01.ane.0000253510.00213.05 17242101

[B13] De Carolis S. Tabacco S. Rizzo F. Giannini A. Botta A. Salvi S. (2018). Antiphospholipid syndrome: an update on risk factors for pregnancy outcome. Autoimmun. Rev. 17 (10), 956–966. 10.1016/j.autrev.2018.03.018 30118899

[B14] Di Martino D. D. Avagliano L. Ferrazzi E. Fusè F. Sterpi V. Parasiliti M. (2022). Hypertensive disorders of pregnancy and fetal growth restriction: clinical characteristics and placental lesions and possible preventive nutritional targets. Nutrients 14 (16), 3276. 10.3390/nu14163276 36014782 PMC9414322

[B15] Dias J. D. Lopez-Espina C. G. Bliden K. Gurbel P. Hartmann J. Achneck H. E. (2020). TEG®6s system measures the contributions of both platelet count and platelet function to clot formation at the site-of-care. Platelets 31 (7), 932–938. 10.1080/09537104.2019.1704713 31878831

[B16] Estcourt L. J. Birchall J. Allard S. Bassey S. J. Hersey P. Kerr J. P. (2017). Guidelines for the use of platelet transfusions. Br. J. Haematol. 176 (3), 365–394. 10.1111/bjh.14423 28009056

[B17] Ferrari B. Peyvandi F. (2020). How I treat thrombotic thrombocytopenic purpura in pregnancy. Blood 136 (19), 2125–2132. 10.1182/blood.2019000962 32797178

[B18] Fitzpatrick K. E. Hinshaw K. Kurinczuk J. J. Knight M. (2014). Risk factors, management, and outcomes of hemolysis, elevated liver enzymes, and low platelets syndrome and elevated liver enzymes, low platelets syndrome. Obstet. Gynecol. 123 (3), 618–627. 10.1097/AOG.0000000000000140 24499757

[B19] Fogerty A. E. (2018). Thrombocytopenia in pregnancy: mechanisms and management. Transfus. Med. Rev. 32 (4), 225–229. 10.1016/j.tmrv.2018.08.004 30177431

[B20] Fogerty A. E. (2024). ITP in pregnancy: diagnostics and therapeutics in 2024. Hematol. Am. Soc. Hematol. Educ. Program 2024 (1), 685–691. 10.1182/hematology.2024000595 39643994 PMC11665594

[B21] Fogerty A. E. Kuter D. J. (2024). How I treat thrombocytopenia in pregnancy. Blood 143 (9), 747–756. 10.1182/blood.2023020726 37992219

[B22] George J. N. Nester C. M. McIntosh J. J. (2015). Syndromes of thrombotic microangiopathy associated with pregnancy. Hematol. Am. Soc. Hematol. Educ. Program 2015, 644–648. 10.1182/asheducation-2015.1.644 26637783

[B23] Gernsheimer T. James A. H. Stasi R. (2013). How I treat thrombocytopenia in pregnancy. Blood 121 (1), 38–47. 10.1182/blood-2012-08-448944 23149846

[B24] Gerth J. Schleussner E. Kentouche K. Busch M. Seifert M. Wolf G. (2009). Pregnancy-associated thrombotic thrombocytopenic purpura. Thromb. Haemost. 101 (2), 248–251. 10.1160/th07-12-0739 19190806

[B25] Govindappagari S. Moyle K. Burwick R. M. (2020). Mild thrombocytopenia and postpartum hemorrhage in nulliparous women with term, singleton, vertex deliveries. Obstet. Gynecol. 135 (6), 1338–1344. 10.1097/AOG.0000000000003861 32459425

[B26] Greinacher A. Selleng S. (2016). How I evaluate and treat thrombocytopenia in the intensive care unit patient. Blood 128 (26), 3032–3042. 10.1182/blood-2016-09-693655 28034871

[B27] Guillet S. Loustau V. Boutin E. Zarour A. Comont T. Souchaud-Debouverie O. (2023). Immune thrombocytopenia and pregnancy: an exposed/nonexposed cohort study. Blood 141 (1), 11–21. 10.1182/blood.2022017277 36054922 PMC10644036

[B28] Haile K. Kebede S. Abera T. Timerga A. Mose A. (2022). Thrombocytopenia among pregnant women in Southwest Ethiopia: burden, severity, and predictors. J. Blood Med. 13, 275–282. 10.2147/JBM.S365812 35651987 PMC9150712

[B29] Huang J. Zeng B. Li X. Huang M. Zhan R. (2020). Comparative Study of the clinical application of 2 bleeding grading systems for pregnant women with immune thrombocytopenia. Clin. Appl. Thromb. Hemost. 26, 1076029620910790. 10.1177/1076029620910790 32392082 PMC7370566

[B30] Jin J. Xu X. Hou L. Hou Y. Li J. Liang M. (2022). Thrombocytopenia in the first trimester predicts adverse pregnancy outcomes in obstetric antiphospholipid syndrome. Front. Immunol. 13, 971005. 10.3389/fimmu.2022.971005 36059524 PMC9433896

[B31] Joly B. S. Coppo P. Veyradier A. (2017). Thrombotic thrombocytopenic purpura. Blood 129 (21), 2836–2846. 10.1182/blood-2016-10-709857 28416507

[B32] Kam P. C. Thompson S. A. Liew A. C. (2004). Thrombocytopenia in the parturient. Anaesthesia 59 (3), 255–264. 10.1111/j.1365-2044.2004.03576.x 14984524

[B33] Kaufman R. M. Djulbegovic B. Gernsheimer T. Kleinman S. Tinmouth A. T. Capocelli K. E. (2015). Platelet transfusion: a clinical practice guideline from the AABB. Ann. Intern Med. 162 (3), 205–213. 10.7326/M14-1589 25383671

[B34] Kelton J. G. (2002). Idiopathic thrombocytopenic purpura complicating pregnancy. Blood Rev. 16 (1), 43–46. 10.1054/blre.2001.0181 11913994

[B36] Li J. Gao Y. H. Su J. Zhang L. Sun Y. Li Z. Y. (2022). Diagnostic ideas and management strategies for thrombocytopenia of unknown causes in pregnancy. Front. Surg. 9, 799826. 10.3389/fsurg.2022.799826 35465428 PMC9019731

[B37] Liu L. Sun D. (2019). Pregnancy outcomes in patients with primary antiphospholipid syndrome: a systematic review and meta-analysis. Med. Baltim. 98 (20), e15733. 10.1097/MD.0000000000015733 31096533 PMC6531250

[B63] Liu X. Wang H. Huang C. Meng Z. Zhang W. Li Y. (2019). Association between platelet distribution width and serum uric acid in Chinese population. Biofactors 45 (3), 326–334. 10.1002/biof.1491 30697838

[B38] Macafee B. Campbell J. P. Ashpole K. Cox M. Matthey F. Acton L. (2012). Reference ranges for thromboelastography (TEG(®)) and traditional coagulation tests in term parturients undergoing caesarean section under spinal anaesthesia. Anaesthesia 67 (7), 741–747. 10.1111/j.1365-2044.2012.07101.x 22486761

[B39] Martin J. N. Jr. Bailey A. P. Rehberg J. F. Owens M. T. Keiser S. D. May W. L. (2008). Thrombotic thrombocytopenic purpura in 166 pregnancies: 1955-2006. Am. J. Obstet. Gynecol. 199 (2), 98–104. 10.1016/j.ajog.2008.03.011 18456236

[B40] McCrae K. R. (2010). Thrombocytopenia in pregnancy. Hematol. Am. Soc. Hematol. Educ. Program 2010, 397–402. 10.1182/asheducation-2010.1.397 21239825

[B41] Mithoowani S. Cervi A. Shah N. Ejaz R. Sirotich E. Barty R. (2020). Management of major bleeds in patients with immune thrombocytopenia. J. Thromb. Haemost. 18 (7), 1783–1790. 10.1111/jth.14809 32219982

[B42] Mol B. W. J. Roberts C. T. Thangaratinam S. Magee L. A. de Groot C. J. M. Hofmeyr G. J. (2016). Pre-eclampsia. Lancet 387 (10022), 999–1011. 10.1016/S0140-6736(15)00070-7 26342729

[B44] Myers B. (2012). Diagnosis and management of maternal thrombocytopenia in pregnancy. Br. J. Haematol. 158 (1), 3–15. 10.1111/j.1365-2141.2012.09135.x 22551110

[B45] Neunert C. Terrell D. R. Arnold D. M. Buchanan G. Cines D. B. Cooper N. (2019). American society of hematology 2019 guidelines for immune thrombocytopenia. Blood Adv. 3 (23), 3829–3866. 10.1182/bloodadvances.2019000966 31794604 PMC6963252

[B46] Park Y. H. (2022). Diagnosis and management of thrombocytopenia in pregnancy. Blood Res. 57 (S1), 79–85. 10.5045/br.2022.2022068 35483931 PMC9057658

[B47] Parnas M. Sheiner E. Shoham-Vardi I. Burstein E. Yermiahu T. Levi I. (2006). Moderate to severe thrombocytopenia during pregnancy. Eur. J. Obstet. Gynecol. Reprod. Biol. 128 (1-2), 163–168. 10.1016/j.ejogrb.2005.12.031 16533554

[B48] Pishko A. M. Marshall A. L. (2022). Thrombocytopenia in pregnancy. Hematol. Am. Soc. Hematol. Educ. Program 2022 (1), 303–311. 10.1182/hematology.2022000375 36485110 PMC9820693

[B49] Pishko A. M. Levine L. D. Cines D. B. (2020). Thrombocytopenia in pregnancy: diagnosis and approach to management. Blood Rev. 40, 100638. 10.1016/j.blre.2019.100638 31757523

[B50] Provan D. Arnold D. M. Bussel J. B. Chong B. H. Cooper N. Gernsheimer T. (2019). Updated international consensus report on the investigation and management of primary immune thrombocytopenia. Blood Adv. 3 (22), 3780–3817. 10.1182/bloodadvances.2019000812 31770441 PMC6880896

[B51] Reese J. A. Peck J. D. Deschamps D. R. McIntosh J. J. Knudtson E. J. Terrell D. R. (2018). Platelet counts during pregnancy. N. Engl. J. Med. 379 (1), 32–43. 10.1056/NEJMoa1802897 29972751 PMC6049077

[B52] Rodeghiero F. Stasi R. Gernsheimer T. Michel M. Provan D. Arnold D. M. (2009). Standardization of terminology, definitions and outcome criteria in immune thrombocytopenic purpura of adults and children: report from an international working group. Blood 113 (11), 2386–2393. 10.1182/blood-2008-07-162503 19005182

[B53] Rottenstreich A. Israeli N. Levin G. Rottenstreich M. Elchalal U. Kalish Y. (2018). Clinical characteristics, neonatal risk and recurrence rate of gestational thrombocytopenia with platelet count <100 × 10(9)/L. Eur. J. Obstet. Gynecol. Reprod. Biol. 231, 75–79. 10.1016/j.ejogrb.2018.10.026 30340119

[B54] Saha P. Stott D. Atalla R. (2009). Haemostatic changes in the puerperium '6 weeks postpartum' (HIP Study) - implication for maternal thromboembolism. Bjog 116 (12), 1602–1612. 10.1111/j.1471-0528.2009.02295.x 19681851

[B55] Sharma S. K. Philip J. (1997). The effect of anesthetic techniques on blood coagulability in parturients as measured by thromboelastography. Anesth. Analg. 85 (1), 82–86. 10.1097/00000539-199707000-00015 9212127

[B56] Smock K. J. Perkins S. L. (2014). Thrombocytopenia: an update. Int. J. Lab. Hematol. 36 (3), 269–278. 10.1111/ijlh.12214 24750673

[B57] Snow L. Knapp G. Llaneza A. J. Sowdagar S. Khawandanah M. O. Vesely S. (2023). Current management of pregnant persons with ITP: Systematic literature review. Blood 142 (Suppl. 1), 3957. 10.1182/blood-2023-191326

[B58] Spiezia L. Bogana G. Campello E. Maggiolo S. Pelizzaro E. Carbonare C. D. (2015). Whole blood thromboelastometry profiles in women with preeclampsia. Clin. Chem. Lab. Med. 53 (11), 1793–1798. 10.1515/cclm-2014-1128 25803079

[B59] Stanworth S. J. Shah A. (2022). How I use platelet transfusions. Blood 140 (18), 1925–1936. 10.1182/blood.2022016558 35926105

[B60] Thomas M. R. Robinson S. Scully M. A. (2016). How we manage thrombotic microangiopathies in pregnancy. Br. J. Haematol. 173 (6), 821–830. 10.1111/bjh.14045 27019232

[B61] Townsley D. M. (2013). Hematologic complications of pregnancy. Semin. Hematol. 50 (3), 222–231. 10.1053/j.seminhematol.2013.06.004 23953339 PMC3748382

[B62] Ushida T. Kotani T. Moriyama Y. Imai K. Nakano-Kobayashi T. Kinoshita F. (2021). Platelet counts during normal pregnancies and pregnancies complicated with hypertensive disorders. Pregnancy Hypertens. 24, 73–78. 10.1016/j.preghy.2021.02.013 33714072

[B64] van der Lugt N. M. van Kampen A. Walther F. J. Brand A. Lopriore E. (2013). Outcome and management in neonatal thrombocytopenia due to maternal idiopathic thrombocytopenic purpura. Vox Sang. 105 (3), 236–243. 10.1111/vox.12036 23782272

[B65] Veneri D. Franchini M. Randon F. Nichele I. Pizzolo G. Ambrosetti A. (2009). Thrombocytopenias: a clinical point of view. Blood Transfus. 7 (2), 75–85. 10.2450/2008.0012-08 19503627 PMC2689060

[B66] Vizioli L. Muscari S. Muscari A. (2009). The relationship of mean platelet volume with the risk and prognosis of cardiovascular diseases. Int. J. Clin. Pract. 63 (10), 1509–1515. 10.1111/j.1742-1241.2009.02070.x 19769707

[B67] Vreede A. P. Bockenstedt P. L. McCune W. J. Knight J. S. (2019). Cryptic conspirators: a conversation about thrombocytopenia and antiphospholipid syndrome. Curr. Opin. Rheumatol. 31 (3), 231–240. 10.1097/BOR.0000000000000595 30747734 PMC6455093

[B68] Walle M. Arkew M. Asmerom H. Tesfaye A. Getu F. (2023). The diagnostic accuracy of mean platelet volume in differentiating immune thrombocytopenic purpura from hypo-productive thrombocytopenia: a systematic review and meta-analysis. PLoS One 18 (11), e0295011. 10.1371/journal.pone.0295011 38033118 PMC10688894

[B69] Wang X. Xu Y. Luo W. Feng H. Luo Y. Wang Y. (2017). Thrombocytopenia in pregnancy with different diagnoses: differential clinical features, treatments, and outcomes. Med. Baltim. 96 (29), e7561. 10.1097/MD.0000000000007561 28723784 PMC5521924

[B70] Wang W. Long K. Deng F. Ye W. Zhang P. Chen X. (2021). Changes in levels of coagulation parameters in different trimesters among Chinese pregnant women. J. Clin. Lab. Anal. 35 (4), e23724. 10.1002/jcla.23724 33543804 PMC8059730

[B71] Woudstra D. M. Chandra S. Hofmeyr G. J. Dowswell T. (2010). Corticosteroids for HELLP (hemolysis, elevated liver enzymes, low platelets) syndrome in pregnancy. Cochrane Database Syst. Rev. (9), Cd008148. 10.1002/14651858.CD008148.pub2 20824872 PMC4171033

[B72] Xie X. Wang M. Lu Y. Zeng J. Wang J. Zhang C. (2021). Thromboelastography (TEG) in normal pregnancy and its diagnostic efficacy in patients with gestational hypertension, gestational diabetes mellitus, or preeclampsia. J. Clin. Lab. Anal. 35 (2), e23623. 10.1002/jcla.23623 33067885 PMC7891543

[B73] Xu J. Tan L. N. Li L. X. Qiao G. Y. (2024). Case report of thrombotic thrombocytopenic purpura during pregnancy with a review of the relevant research. Med. Baltim. 103 (20), e38112. 10.1097/MD.0000000000038112 38758904 PMC11098172

[B74] Yan M. Malinowski A. K. Shehata N. (2016). Thrombocytopenic syndromes in pregnancy. Obstet. Med. 9 (1), 15–20. 10.1177/1753495X15601937 27512485 PMC4950432

[B75] Young B. Levine R. J. Salahuddin S. Qian C. Lim K. H. Karumanchi S. A. (2010). The use of angiogenic biomarkers to differentiate non-HELLP related thrombocytopenia from HELLP syndrome. J. Matern. Fetal Neonatal Med. 23 (5), 366–370. 10.1080/14767050903184207 19701867 PMC3132879

[B76] Yuce T. Acar D. Kalafat E. Alkilic A. Cetindag E. Soylemez F. (2014). Thrombocytopenia in pregnancy: do the time of diagnosis and delivery route affect pregnancy outcome in parturients with idiopathic thrombocytopenic purpura? Int. J. Hematol. 100 (6), 540–544. 10.1007/s12185-014-1688-6 25293555

[B77] Zhou Y. Reilly S. D. Gangaraju R. Reddy V. V. B. Marques M. B. (2017). An unusual presentation of thrombotic thrombocytopenic purpura. Am. J. Med. 130 (8), e323–e326. 10.1016/j.amjmed.2017.04.022 28528921

